# Novel Insights into Cr(VI)-Induced Rhamnolipid Production and Gene Expression in *Pseudomonas aeruginosa* RW9 for Potential Bioremediation

**DOI:** 10.4014/jmb.2406.06034

**Published:** 2024-07-19

**Authors:** Fatini Mat Arisah, Norhayati Ramli, Hidayah Ariffin, Toshinari Maeda, Mohammed Abdillah Ahmad Farid, Mohd Zulkhairi Mohd Yusoff

**Affiliations:** 1 Department of Bioprocess Technology, Faculty of Biotechnology and Biomolecular Sciences, Universiti Putra Malaysia, Serdang 43400, Selangor, Malaysia; 2 Laboratory of Biopolymer and Derivatives, Institute of Tropical Forestry and Forest Products (INTROP), Universiti Putra Malaysia, 43400 UPM Serdang, Selangor, Malaysia; 3 Department of Biological Functions Engineering, Graduate School of Life Science and Systems Engineering, Kyushu Institute of Technology, 2-4 Hibikino, Wakamatsu-ku, Kitakyushu 808-0196, Japan

**Keywords:** Bioremediation, rhamnolipid, chromium hexavalent, *Pseudomonas aeruginosa*

## Abstract

Rhamnolipid (RL) is renowned for its efficacy in bioremediating several types of organic and metal contaminants. Nevertheless, there has been a scarcity of studies specifically examining the relationship between this substance and metals, especially in terms of their impact on RL formation and the underlying interaction processes. This study addresses this gap by investigating the RL mechanism in Cr (VI) remediation and evaluating its effect on RL production in *Pseudomonas aeruginosa* RW9. In this study, *P. aeruginosa* RW9 was grown in the presence of 10 mg l^-1^ Cr (VI). We monitored RL yield, congeners distribution, and their ratios, as well as the transcriptional expression of the RL-encoded genes: *rhlA*, *rhlB*, and *rhlC*. Our results revealed that RL effectively reduced Cr (VI) to Cr (III), with RL yield increasing threefold, although with a slight delay in synthesis compared to control cells. Furthermore, Cr (VI) exposure induced the transcriptional expression of the targeted genes, leading to a significant increase in di-RL production. The findings confirm that Cr (VI) significantly impacts RL production, altering its structural compositions and enhancing the transcriptional expression of RL-encoded genes in *P. aeruginosa* RW9. This study represents a novel exploration of Cr (VI)'s influence on RL production, providing valuable insights into the biochemical pathways involved and supporting the potential of RL in Cr (VI) bioremediation.

## Introduction

*Pseudomonas aeruginosa* is an extremely versatile, rod-shaped Gram-negative bacterium that is ubiquitous in the environment. Due to its efficiency, the bacterium is among the most studied for the remediation of various pollutants, both organic [33] and inorganic [27, 40] substances. Its efficacy is attributed to various removal mechanisms such as cell membrane adsorption, enzymatic as well as non-enzymatic reduction, and secretion of metabolites [52]. However, its classification as an opportunistic pathogen hinders its direct application in large-scale wastewater remediation. To overcome these challenges, research has shifted focus to its glycolipid biosurfactant, rhamnolipid (RL). Despite extensive research, the specific physiological significance of RL remains not fully understood.

RL is known to play a significant role in the survivability of *P. aeruginosa* by contributing to virulence activation, which benefits the colony as a whole [13]. Previous studies have highlighted RL’s involvement in biofilm formation, swarming motility on semi-solid surfaces, and enhanced bioavailability of hydrophobic substances like long-chain hydrocarbons, demonstrating its adaptability to various environmental conditions [3, 36]. However, less attention has been given to RL's interaction with metals, specifically regarding their effects on RL production and its interaction with the produced glycolipid.

Recent research underscores the pivotal role of rhamnolipid (RL) in metal ion interactions, serving as a resistance mechanism in RL-producing strains. The multifunctional nature of RL contributes to their effectiveness in protecting bacterial cells from metal toxicity and enhancing metal sequestration in contaminated environments. González *et al*. [20] demonstrated that RL significantly decrease Cu(II) adsorption on the bacterial cell wall, acting as a protective barrier by preventing Cu(II) ions from complexing with cell wall components. Similarly, Kenney and Fein [30] found that RL production reduced Cd(II) adsorption on bacterial surfaces, highlighting the specificity of RL in mitigating metal ion toxicity. Spormann *et al*. [50] further evidenced RL's role as effective biosurfactants, capable of binding and sequestering Zn(II) and Pb(II) ions through glycolipid interactions, thus preventing the formation of insoluble metal complexes that could damage bacterial cells. Izrael-Živković *et al*. [27] suggested that RL improve metal ion complexation, with 25% of Cd(II) binding to the glycolipid, indicating a significant enhancement in the detoxification capacity.

RL-producing bacteria have the ability to tolerate metals such as Ca, Cd, Cu, Co, Cr, Li, Mn, Pb, Zn, and Se (with the exception of Hg), which highlights its use in phytoremediation [48, 53]. Additionally, the research conducted on Cd stress showed that RL had a function in lowering the buildup of intracellular Cd (II) to relieve stress in the RL-producing bacteria. Moreover, the RL that were synthesized displayed modified physicochemical characteristics when exposed to high levels of Cd stress [57]. The production of RL facilitates the stabilization and removal of heavy metals, contributing to the overall health and sustainability of the ecosystem. These combined findings illustrate the multifaceted role of RL in bacterial resistance to heavy metals, functioning both as biosurfactants that sequester metal ions and as protective agents that prevent harmful interactions with cell walls.

To date, few metal species have been explored for their effect on RL production by *P. aeruginosa*, with Fe(II) and Fe(III) being the most studied micronutrients [34, 47]. Limiting the availability of Fe (II)/Fe (III) enhances RL production while concurrently suppressing cell growth. This phenomenon is not unique to iron ions as it has also been observed with other hazardous divalent ions such as Cd(II) [27] and Pb(II) [50]. Studies on iron (Fe) influence on RL gene expression suggest a regulatory association between RL production and metal concentration [47] involving the transcriptional regulation of key RL-encoded genes, *rhlA*, *rhlB*, and *rhlC* [14]. These genes are crucial in RL biosynthesis, regulated by quorum sensing through acyl-homoserine lactone [36]. Various metals have diverse effects on the formation of RL. For instance, it has been shown that the presence of Cd (II) increases the formation of RL in *P. aeruginosa*. This results in continued production throughout the late stationary growth phase and an increase in the proportion of di-RL to mono-RL congeners [39]. Based on these findings, metals indeed can significantly influence RL production and the distribution of its congeners, thereby explaining observed differences in metal affinity.

In our previous work, we elucidated the effect of Cr(VI) on the growth of *P. aeruginosa* RW9 and its removal efficiency, demonstrating the bacterium’s potential in Cr(VI) remediation [4]. We also found that it produced glycolipid biosurfactant aiding in ion sequestration. To confirm our hypothesis that the glycolipid biosurfactant aids in Cr (VI) reduction, we quantified Cr (III) concentration and Cr (VI) removal percentage by the produced RL. We also quantified, extracted, and characterized the RL produced, assessing its congeners’ distribution and the effect of Cr (VI) on its structural conformation. Furthermore, we quantified the transcriptional expression of *rhlA*, *rhlB*, and *rhlC* to understand Cr (VI)'s impact on RL biosynthesis.

Given its carcinogenic and mutagenic properties, the efficient removal of Cr (VI) from industrial effluents is critically important. RL has gained attention as a bioremediation tool for a variety of organic and metal pollutants [46]. Still, barely any studies have looked into the way *P. aeruginosa* and Cr (VI) interact with one another to affect RL formation and the mechanisms by which this happens. This study represents a distinctive exploration, broadening the understanding of Cr (VI)'s influence on the transcriptomic and metabolomic levels of RL production, thereby supporting RL's pivotal role in Cr (VI) remediation. Our findings reveal Cr (VI)'s capability to modulate the biochemical pathway of RL, suggesting its potential application as an additional nutrient in RL production media. This insight facilitates strategic manipulation of production processes, adding a valuable dimension to the existing knowledge base in metals bioremediation.

## Materials and Methods

### Bacterial Strain and Cultivation Media

*Pseudomonas aeuginosa* RW9 was obtained from the Environmental Biotechnology Laboratory, Universiti Putra Malaysia [4]. To assess tolerance levels, we used another bacterial strain called *Bacillus* sp. L14 is known for its high tolerance and effectiveness in removing Cr(VI) based on prior research [22]. It was purchased from the German Collection of Microorganisms and Cell Cultures GmbH (Germany). For the isolation of individual bacterial colonies, the bacterial strains were streaked onto nutrient agar plates and incubated at 30°C for 24 h. A single colony from each strain was transferred into 50 ml of nutrient broth (NB) containing 5 g l^-1^ of peptone and 3g l^-1^ of yeast extract. The cells were cultured in a shaking incubator at 150 ×*g* until they reached the late exponential phase, characterized by a final optical density (OD) between 1.8 and 2.0. Each experiment was seeded with a 10% (v/v) inoculum.

### Growth Assessment and Cr (VI) Removal Study

For the growth assessment and Cr(VI) removal study, 10 mg l^-1^ of Cr(VI) was supplemented to the media as a continuity to our previous work [4]. The concentration was shown to exhibit the highest Cr (VI) removal with a lesser effect on cell growth (see Fig. S1). Media without Cr (VI) served as the control. The samples were taken at predetermined time intervals, and centrifuged at 10,000 ×*g*, 4°C for 20 min to obtain the supernatant. The obtained pellet was washed using 0.85% NaCl solution and resuspended before optical density measurement at 600 nm using a UV/visible spectrophotometer Hitachi UV 2900 (Hitachi Ltd., Japan). The Cr (VI) concentration was determined using the HACH kit (ChromaVer Powder USA) which used the 1,5-diphenylcarbazide method according to its protocol. To confirm the reduction reaction as the Cr (VI) removal mechanism, the presence of trivalent chromium (Cr (III)) was detected by determining the total chromium concentration. The chromium total powder pillow (HACH, USA) following Method 8024; the alkaline hypobromite oxidation method (APHA, 2005), was used in the detection. The intensity of the violet coloration was measured at an absorbance of 540 nm by using a UV/visible spectrophotometer (Hitachi UV 2900, Hitachi Ltd., Japan). The Cr (III) was determined mathematically by subtracting the concentration of Cr (VI) from the total Cr concentration obtained.

### Determination of Dominant Metabolite in Cr (VI) Removal

To confirm the dominant metabolite that removed Cr (VI), a removal experiment using spent media was conducted. The spent media was obtained by removing cells through centrifugation at 10,000 ×*g*, 4°C for 20 min after 24 h of incubation in NB. The supernatant was collected, and heat treated at 65°C for 30 min to denature any remaining residual proteins or reductases that might be present in the supernatant. The effect of reductase was chosen to be eliminated considering its lower tolerance to temperature. The heated supernatant was cooled at room temperature and filtered through a 0.22 μm PTFE syringe filter (BIOFLOW, Malaysia) for sterilization. It was added to NB with 10 mg l^-1^ Cr(VI) at 10% (v/v) to mimic the conditions as in the removal experiments by the cells [4]. The sample was taken out every 4 h as in Section 2.2 to assess for Cr (VI) removal by RL. The Cr (VI) extracellular removal obtained was calculated by subtracting the detected Cr (VI) concentration from the initial Cr (VI) concentration. The Cr (VI) removal by respective metabolites was calculated by using the equation as follows.

$${\text{Cr(VI)}}_{\text{total removal }}={\text{Cr(VI)}}_{\text{RL}}+{\text{Cr(VI)}}_{\text{reductase}}$$ (1)

Thus,

$${\text{Cr(VI)}}_{\text{total removal }}-{\text{Cr(VI)}}_{\text{RL}}={\text{Cr(VI)}}_{\text{reductase}}$$ (2)

### RL Quantification

The fermentation was conducted for 24 h with a sampling time of 4 h intervals. The cultures were incubated at 30°C and 150 ×*g* shaking. At each time interval, the samples were harvested by 10,000 ×*g* centrifugation at 4°C and prepared as suggested by Smyth *et al*. [49]. The supernatant collected was derivatized by using 2- bromoacetophenone and triethylamine (Et_3_N) which were dissolved in acetonitrile (CH_3_CN) at a molar ratio of 1:4:2 (samples (glycolipid): 2-bromoaceto-phenone: Et_3_N). The samples were heated at 80°C for 1 h and filtered through a 0.22 μm PTFE syringe filter (BIOFLOW, Malaysia) once cooled. A high-performance liquid chromatography, HPLC-VW 1200 Series (Agilent Technologies, USA) was used to quantify the RL in the samples. The VW detection was set at 244 nm using CH_3_CN (mobile phase A) and 3.3 mM H_3_PO_4_ (mobile phase B) connected to a Zorbax ODS C18 column (250 mm × 4.6 mm × 5 μm) (Agilent Technologies). The gradient conditions of both mobile phases were set as follows: 50% A and 50% B for 3 min, then to 100% A over 19 min and held for 5 min, followed by a change to 50% A over 3 min and held for 10 min. An injection volume of 50 μl with a flow rate of 1.0 ml min^-1^ was set. The peak area obtained for each sample was compared to a standard curve of commercial RL (Sigma-Aldrich, USA) that has similarly been derivatized for quantification. The quantity of the RL was measured in mg l^-1^.

### RL Recovery

A 1 L working volume fermentation was carried out for RL recovery. At the end of incubation, the biosurfactant was extracted by using acid precipitation coupled with a solvent extraction method with slight modification as suggested by Smyth *et al*. [49]. The whole broth was centrifuged at 10,000 ×*g* for 10 min to remove the cells. The supernatant was acidified with concentrated H_3_PO_4_ to pH 2 [59] and transferred to a separating funnel. Later, an ethyl acetate at a 1:1 ratio was added and shaken vigorously [49]. Two distinct layers were allowed to form: the upper layer comprised the extracted biosurfactant in ethyl acetate, while the lower layer consisted of NB. The solvent extraction step was repeated three times to ensure all RL was extracted from the NB. The top aqueous layer was transferred to a separate flask and 0.5 g of MgSO_4_ was added for every 100 ml of ethyl acetate to remove traces of water.

The mixture was then filtered and evaporated by using rotary evaporation. The extract obtained was further dried using nitrogen gas before being purified using StrataR SI-1 Silica (55 μm, 70 A) (Phenomenex, USA). The solid phase extraction (SPE) column was connected to a Rocker 300 vacuum pump (Rocker Scientific, Taiwan) to suck the solution from the column. It was first conditioned with chloroform followed by deionized distilled water [17]. The crude RL was dissolved in 1.5 ml of chloroform and loaded into the column. It was left to run through the column before adding 2 ml of 1% NaOH to wash the sample. A continuous washing step was done until there were no color changes observed at the output of the column [6]. The eluted purified RL product was dried in a fume hood and collected to be used in characterization analyses.

### RL Characterization Analyses

**Nuclear magnetic resonance (NMR) analysis.** Purified RL of 6 mg was dissolved in deuterated chloroform [29] before being subjected to VnmrS 500MHz NMR (Varian, UK) for a 1D NMR analysis (^1^H and ^13^C experiments). In both NMR experiments, tetramethylsilane (TMS) and deuterated chloroform were used as internal standard and solvent, respectively. The chromatogram obtained was analyzed using MestReNova software by Mestrelab Research (Santiago, Spain).

**Fourier transform infrared spectroscopy (FTIR) analysis.** The functional groups present in the samples were identified using a diamond single reflection attenuated total reflectance Fourier transform infrared spectroscopy (FTIR-ATR) (IRAffinity-1S FTIR, Shimadzu, Japan). The samples were scanned at a spectral resolution of 4 cm^-1^ and wave number range from 4,000 to 400 cm^-1^. The spectra obtained were compared with those reported in previous works [38, 45].

**Liquid chromatograph-mass spectrometry analysis (LC-MS).** The RL congener distribution was interpreted using Ultimate 3000 Series with Q Exactive Focus Mass Spectrometer (Thermo Fisher Scientific, USA) equipped with a C18 reversed-phase column (Hypersil Gold aQ, 100 × 2.1 mm, 1.9 μm, Thermo Fisher Scientific, USA). The photodiode array (PDA) detector was coupled with an electrospray ionization interface (ESI). The negative ion mode producing [M–H] pseudomolecular ions was used to detect the samples with the scanning mass range from 150 to 2,000 m/z (mass to charge ratio). The parameters for the detection were set following Wittgens *et al*. [55] with slight modifications. The sample injection volume was set to 5 μl with a flow rate of 0.3 ml min^-1^ throughout the 30-min run. Formic acid with a concentration of 0.1% was added to deionized water (mobile phase A) and acetonitrile (mobile phase B), respectively. The gradient conditions were set as follows: 40% B at 0 min, increased to 95% B at 25 min before decreasing to 40% from 27 to 30 min. RL congeners were identified based on their expected m/z and their relative abundance was calculated using the obtained area for each congener.

### Transcriptional Expression Study

**Genomic DNA extraction.** An overnight culture of the bacterium was harvested by centrifugation (Centrifuge 5418 Eppendorf, Germany) at 10,000 ×*g* for 10 min to obtain the pellet. Its genomic DNA extraction was done by using NucleoSpin^TM^ Soil (Macherey-Nagel, Germany) according to the manufacturer`s protocol. The concentration and purity of the extracted DNA were measured using a NanoDrop 2000c spectrophotometer (Thermo Fisher Scientific). The samples were then subjected to gel electrophoresis for integrity assessment. Agarose gel of 1.5%concentration was prepared by dissolving agarose powder (Vivantis, Malaysia) in 1× Tris-acetic acid- EDTA (TAE) buffer. The electrophoresis was run at 110 volts for 30 min using a Sub-Cell GT Horizontal Electrophoresis Systems (Bio-Rad Laboratories, USA). The gel was then viewed under UV light in Gel Doc^TM^ XR System (Bio-Rad Laboratories Inc.). The extracted genomic DNA was stored at -20°C for future use.

**Evaluation and validation of target genes.** Genomic DNA obtained from Section 2.7.1 was sent to a service provider (Neoscience Sdn. Bhd., Petaling Jaya, Malaysia) for Whole Genome Sequencing (WGS). The library was constructed by using MGIEasy FS DNA Library Prep Set (MGI Tech Co. Ltd., China) according to the manufacturer's manual with the DNBSEQ-G400 (MGI Tech Co. Ltd.) as the sequencer. The raw reads obtained were first merged and filtered for low-quality sequences. Low base quality thresholds of 20 and 0.1% of N base were selected for Phread33 parameters. The sequence was assembled using the Microbial Genome Analysis Pipeline (MGAP V1.2.2) and annotated with the reference genome of *P. aeruginosa* PAO1 (https://www.ncbi.nlm.nih.gov/assembly/GCF_000006765.1#/def). The sequence acquired in the fast alignment (FASTA) format was then searched in the Kyoto Encyclopedia of Genes and Genomes (KEGG) database using the Blastkoala tool (https://www.kegg.jp/blastkoala/). The target genes for RL-encoded genes were screened.

The presence of RL-encoded genes; *rhlA*, *rhlB*, *rhlC*, and chromate reductase-encoded gene: *chrR* were confirmed and validated using conventional PCR. DreamTaq PCR Master Mix (Thermo Fisher Scientific) was used to amplify 6 μl template DNA (obtained in Section 2.7.1) with 5 μl of 1 mM of each respective primer in a total volume reaction of 50 μl. The conditions for the amplification were as follows: initial denaturation of 3 min at 94°C followed by 30 cycles of denaturation and annealing for 30 s at 94°C and 55°C, respectively, and an extension step at 72°C for 1 min [58]. The final extension was conducted at 72°C for 5 min. The primer sequence of each target gene is listed in Table 1. Gel electrophoresis was used to validate the size of the PCR product for each primer.

**Quantification of transcriptional rhlA, rhlB, rhlC, and chrR gene expression by quantitative reverse transcriptase polymerase chain reaction (qRT-PCR).** At each sampling point, 2 ml samples were pipetted out from each culture flask and added to RNAlaterR solution (Ambion Inc., USA) with a ratio of 1:2 (sample: RNA later) [39]. The mixture was stored at -80°C. RNA was extracted using the Fastpin Total RNA extraction kit (PureNA, Malaysia) according to the manufacturer’s instructions. Samples taken at the same time point were processed together to minimize biases resulting from experimental procedures. The concentration of the extracted RNA was quantified with a NanoDrop 2000c spectrophotometer (Thermo Fisher Scientific), and its integrity was confirmed by gel electrophoresis. The samples were stored at -80°C for future use.

Before qRT-PCR, the annealing temperature of all target and reference genes was optimized by using conventional gradient PCR (Takara Bio Inc., Japan) with a temperature range of 54 to 64°C with an interval of 2°C. The amplification was conducted under similar conditions as in Section 2.7.2. The amplification and quantification of the target and reference genes were performed by using the QuantiNova SybrGreen RT-PCR kit (Qiagen, Germany) following the protocol provided. Primers for target and reference genes (Table 1) were selected with comparable annealing temperatures to permit amplification of the genes from the same time point on a single 48-well plate of an Applied Biosystem Step-one instrument equipped with StepOne^TM^ Software v2.2.2 (Thermo Fisher Scientific). A mixture of SYBR green master mix, Rox dye, and RT mix of 1 × concentration with 0.5 μM of each primer and 200 ng RNA template was prepared. A non-template reaction was set up to ensure no reagent contamination. Transcript levels of the target genes were quantified as the relative expression of the target gene to the housekeeping genes using the Livak method (see Eq. 3) [37].


$$2^{\left(-\Delta \Delta Ct\right)}$$ (3)

Where,

ΔCt = Ct of target gene – Ct of the housekeeping gene

ΔΔCt = ΔCt of treated sample - ΔCt of the control sample

### Statistical Analysis

The data is presented as mean ± standard deviation. The T-test, Kruskal Wallis, and Mann-Whitney U tests were used to analyze the data following their normality distribution, by using the statistical software IBM SPSS Statistics 20 (IBM, USA). A value of *p* ≤ 0.05 was considered statistically significant. All experiments were conducted in triplicates unless otherwise stated.

## Results and Discussion

### Growth Assessment and Cr (VI) Removal Efficiency

**Fig. 1:**
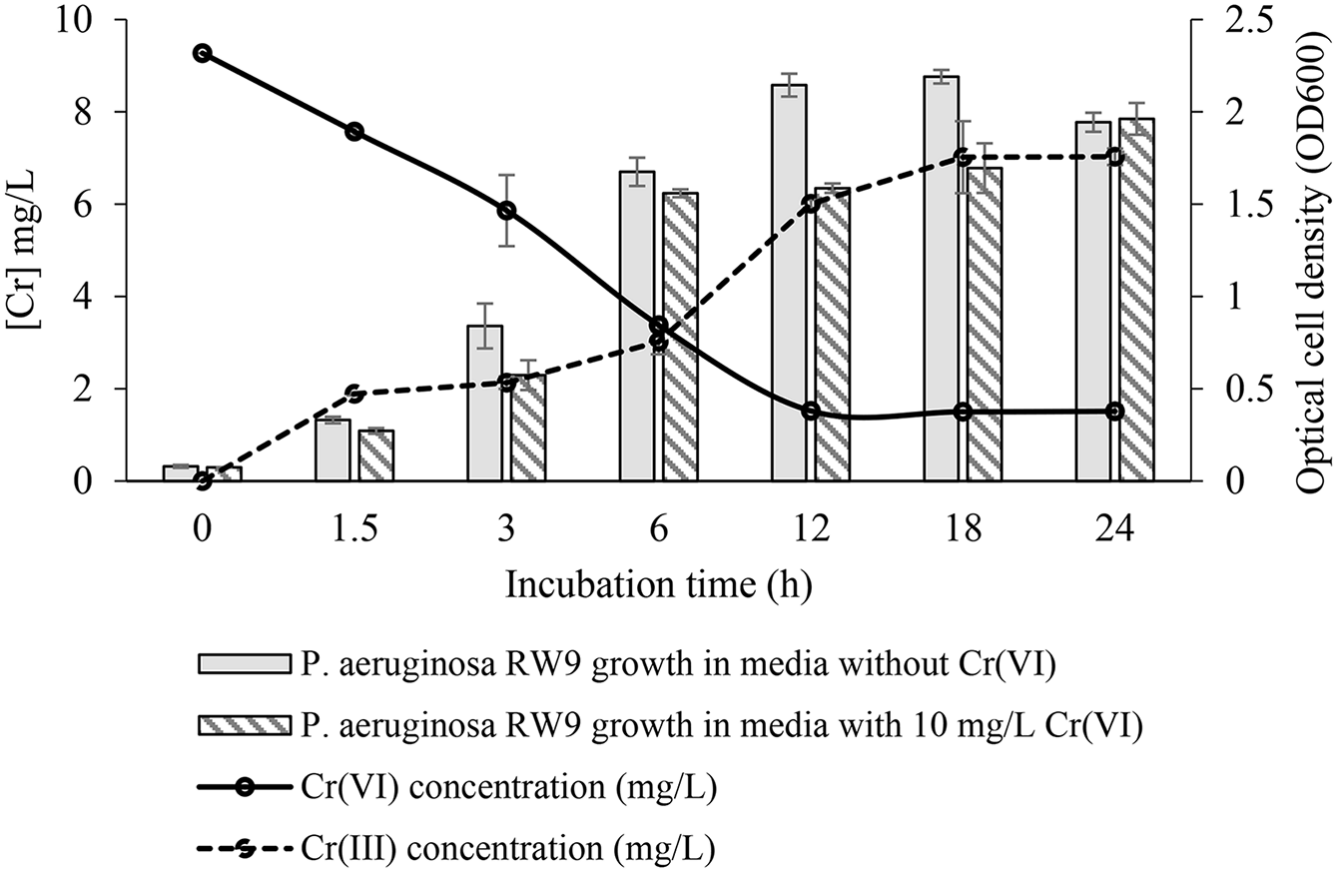
Correlation between the growth profiles of *P. aeruginosa* RW9 and Cr (VI) reduction over 24-h incubation period at 30°C and 150 rpm agitation.

Fig. 1 illustrates that cells cultivated in 10 mg l^-1^ Cr (VI) exhibited significantly slower growth (*p* ≤ 0.05), with a growth rate of 0.54 h^-1^ compared to 0.63 h^-1^ for the control cells. This slower growth rate is attributed to the toxicity associated with radical ions produced as by-products of Cr(VI) reduction [28]. As Cr (VI) is sequentially reduced to Cr (III), reactive molecules such as hydrogen peroxide are generated. These molecules cause oxidative stress in the cells, leading to macromolecular damage and numerous physiological malfunctions [18, 23]. The data shows a steady decrease in Cr (VI) concentration accompanied by a gradual increase in Cr (III) concentration over time, confirming the occurrence of the reduction reaction. Furthermore, the findings demonstrate the effective reducing activity of *P. aeruginosa* RW9, indicating its potential for metal removal beyond mere tolerance to high metal concentrations. The growth-dependent nature of the removal process is evident, as the increase in cell density correlates with a significant Cr (VI) removal rate of 85%.

**Fig. 2:**
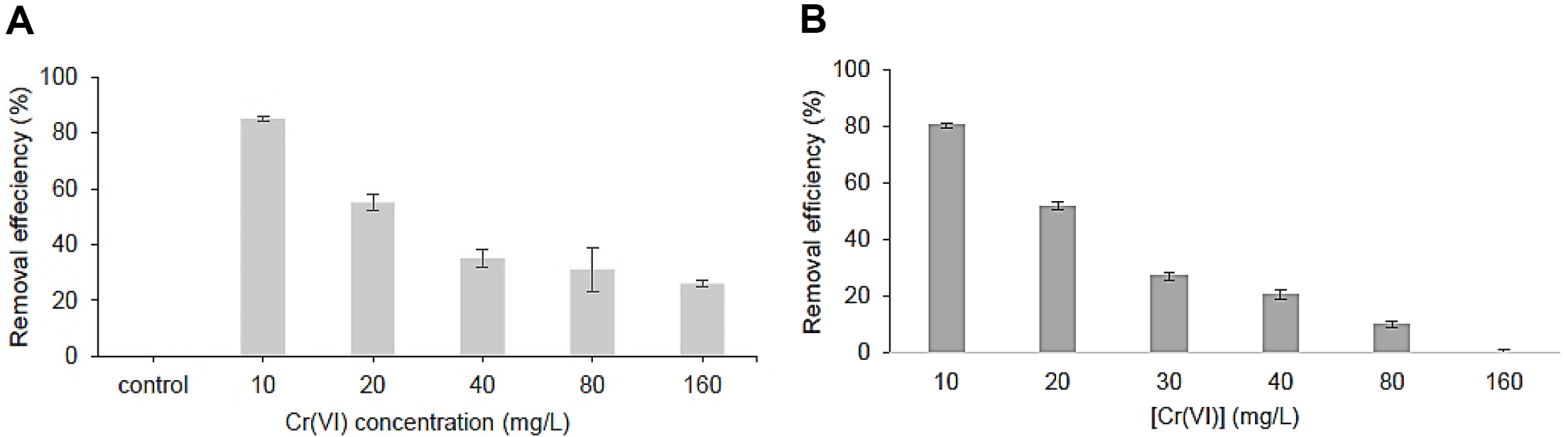
Cr (VI) removal efficiency by (A) *P. aeruginosa* RW9 and (B) *Bacillus* sp. L14 at different Cr (VI) concentrations, under conditions of 30°C and 150 rpm agitation for 24 h in NB.

Despite the *P. aeruginosa* RW9 being able to maintain its functionality up to a concentration of 160 mg l^-1^ without complete loss of ability, Fig. 2 demonstrates a gradual drop in removal efficiency as the Cr (VI) concentration rises, reaching less than 40%. The decrease in cellular function is a result of heightened oxidative stress and damage caused by elevated quantities of toxic Cr (VI) and its reactive derivatives, as previously stated. Moreover, the metabolic demand to counteract this stress redirects resources away from Cr (VI) reduction. Increased concentrations may also restrict the availability of Cr (VI) by causing precipitation or the formation of complexes, hence decreasing efficiency.

In addition, this research conducted a comparison utilizing *Bacillus* sp. L14, which has previously been reported in the literature to have exceptional rates of Cr(VI) removal, with an efficiency of over 90% [5]. Fig. 2 demonstrates that *P. aeruginosa* RW9 exhibited persistent Cr (VI) removal even at higher concentrations of up to 160 mg l^-1^. In contrast, *Bacillus* sp. L14 showed a complete inability to remove Cr (VI) at the same dose. This suggests that *P. aeruginosa* RW9 is very adaptive to chromium-induced DNA damage, using intricate strategies such as extracellular sequestration via rhamnolipid synthesis, as shown in our preliminary research [4].

### Determination of Dominant Metabolite in Cr (VI) Removal and Quantification

**Fig. 3:**
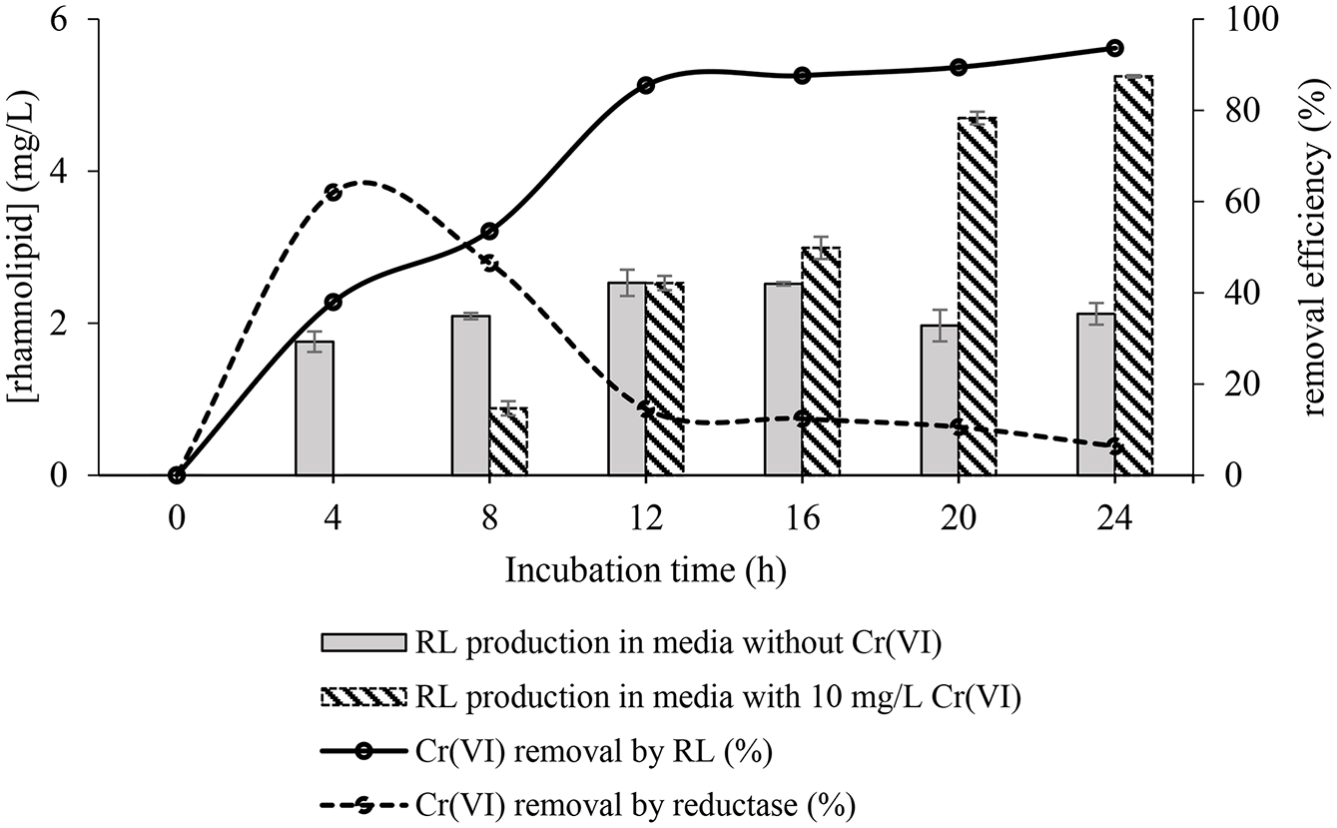
Correlation profile between production of RL and reductase by *P. aeruginosa* RW9 and Cr (VI) removal efficiency over 24 h incubation period at 30°C and 150 rpm agitation.

Our previous investigation has demonstrated that *P. aeruginosa* RW9 primarily employs extracellular sequestration via extracellular reductase and RL to remove Cr(VI) [4]. Within the first 4 h of incubation, reductase was the primary metabolite responsible for reducing Cr (VI) to Cr (III), making up 62.02 ± 0.85% of the total (see Fig. 3). Nevertheless, its significance rapidly decreased from the 4^th^ to the 8^th^ h. Exposure to Cr(VI) has been seen to cause an increase in the production of stress and free radical detoxification proteins [31]. These proteins help in reducing Cr(VI) and neutralizing its radical by-products, protecting cells from oxidative stress [52], as extensively discussed in the latter part of the text. In addition, it was shown that proteins responsible for protein biosynthesis and energy generation were excessively expressed when Cr(VI) was present [31]. The increase in stress response proteins often occurs simultaneously with the suppression of proteins essential to growth. As a result, cells first focus on reducing stress, which causes slower development during the early phases of incubation. During the later phases of incubation, cells successfully overcome stress, leading to a resumption of growth and an increase in metabolite synthesis. Significantly, between the 4^th^ and 8^th^ h, the function of reductase diminishes progressively, being replaced by RL production starting from the 8^th^ h. As the cell density increases, it is expected that there will be a proportional increase in RL production. This hypothesis has been confirmed by a quantitative study [59].

HPLC-UV analysis revealed early production of RL as early as the 4^th^ h in the control sample, with no significant increase observed towards the end of incubation (see Fig. 3). This conducive environment supports RL production, which contributes to the formation of various cellular components, particularly structural units crucial for cell motility [10] and biofilm formation [54]. *P. aeruginosa* often forms multicellular communities [36] initiated by RL-based biofilm formation [54]. Established biofilms create a favorable environment for cell growth, enhancing population expansion by facilitating efficient nutrient distribution [15]. Furthermore, a slow decrease from maximum RL productivity (2.53 ± 0.17 mg l^-1^) towards the end of incubation (1.97 ± 0.2 mg l^-1^) supports the hypothesis regarding RL's role in biofilm formation, suggesting its multifaceted functions beyond resistance or tolerance mechanisms to stressors [10].

In contrast, delayed RL production was observed in cells exposed to Cr (VI), occurring not until the 8^th^ h of incubation. The reduced synthesis of RL under these circumstances could mean an adaptive response to the cellular stress caused by Cr (VI), causing metabolic alterations and altered gene expression of *P. aeruginosa* RW9. In the presence of Cr (VI), which evidenced reduced cell density due to its toxic effects, the bacteria adopt a mechanism to enhance RL production. RL, known for its ability to remediate Cr(VI) through biosorption [11, 12, 42], becomes pivotal in mitigating the effects of Cr(VI) by increasing its production in response to decreased cell density induced by Cr(VI) presence. This adaptive response highlights the multifunctionality of RL, not only serving as a quorum-sensing molecule but also as a key component in the bacterial defense mechanism against environmental stressors like Cr (VI). It is important to note that RL production continues throughout incubation, peaking during the stationary phase (5.25 ± 0.02 mg l^-1^), twice as much as in the control (see Fig. 3). This highlights RL's crucial role in shielding cells from stressors.

Previous research has demonstrated a relationship between cell density and activation of RL regulation systems in *Pseudomonas* [15, 24]. In *Pseudomonas*, the las and rhl systems are quorum-sensing mechanisms tied to cell density [36]. Within the las system, LasR and LasI function interchangeably. LasI is an enzyme that produces a signaling molecule known as 3-oxo-C12-homoserine lactone (3OC12HSL). As the cell density increases, more 3OC12HSL is produced, forming complexes with LasR. LasR, a transcriptional regulator protein, binds to 3OC12HSL. This binding activates or represses the transcription of specific genes, controlling various cellular processes like virulence factor production, biofilm formation, and antibiotic resistance [35]. Together, LasI and LasR form a quorum sensing system, enabling *P. aeruginosa* RW9 to detect and react to changes in population density, coordinating collective behaviors in response to environmental cues.

In the context of RL production, the 3OC12HSL-LasR complex regulates the production of RhlR protein and butanoyl homoserine lactone (C4HSL) [44]. RhlR is a transcriptional regulator protein that is part of the rhl quorum sensing system, which helps bacteria communicate and coordinate their behaviors based on cell density, while C4HSL is a second inducer molecule produced by the enzyme RhlI, which its concentration increases as bacterial cell density rises. When C4HSL levels are high, they bind to RhlR, forming C4HSL-RhlR complexes that trigger the *rhlA* promoter, hence initiating the transcription of genes (*rhlAB* and *rhlC*) for RL synthesis.

### RL Characterization

**Fig. 4:**
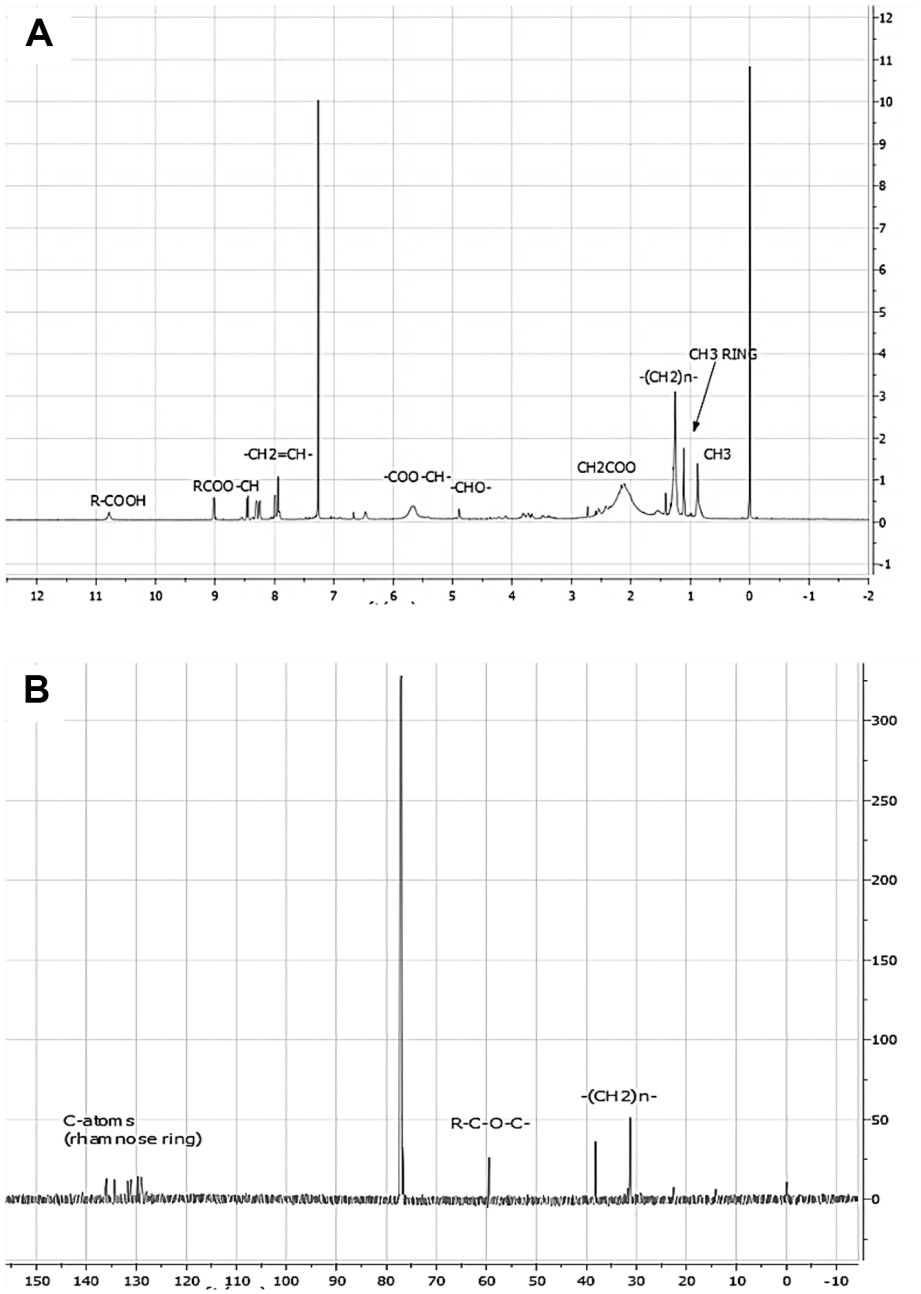
(A) proton NMR and (B) carbon NMR spectra of extracted, purified RL produced by *P. aeruginosa* RW9 after 24 h of cultivation.

A comprehensive 1D (^1^H and ^13^C) NMR experiment was conducted to elucidate the structure of the glycolipid produced. Sharp peaks observed at 0 and 7.20 ppm were attributed to the standard and solvent, respectively (refer to Fig. 4A). The chemical shifts ranging from 0.8 to 2.4 ppm corresponded to the long lipid chains comprising methyl (-CH_3_), alkyl (-CH_2_), and allylic (R=C-CH_3_) groups. Additionally, chemical shifts at 8.0, 9.0, and 10.8 ppm indicated the terminal lipid moiety, corresponding to -CH_2_-CH-, -COO-CH, and -COOH functionalities [19]. The presence of doublet peaks at these chemical shifts, except for -COOH, suggested the existence of a double bond in the bonding of the hydrocarbon lipid chain to the respective functional groups. Furthermore, the rhamnose moiety of the RL was identified at chemical shifts ranging from 3.3 to 5.5 ppm, representing –CH–OH, –O–CH–, CH–O–C, and –COO–CH– bonds of the sugar ring structure. Intense peak signals in the ^13^CNMR spectrum were recorded at 30 to 40 ppm, indicating the presence of methylene group (-CH_2_) of the lipid chain (see Fig. 4B). Moreover, the attachment of the carbon atom of the lipid chain to the oxygen atom of the rhamnose was evidenced by a chemical shift in the range of 60-70 ppm, while peaks in the 120-140 ppm range signified the presence of carbon in the rhamnose rings [51]. As complementary data for the result obtained, FTIR and LC-MS were conducted to confirm the structural conformation of the glycolipid.

**Fig. 5:**
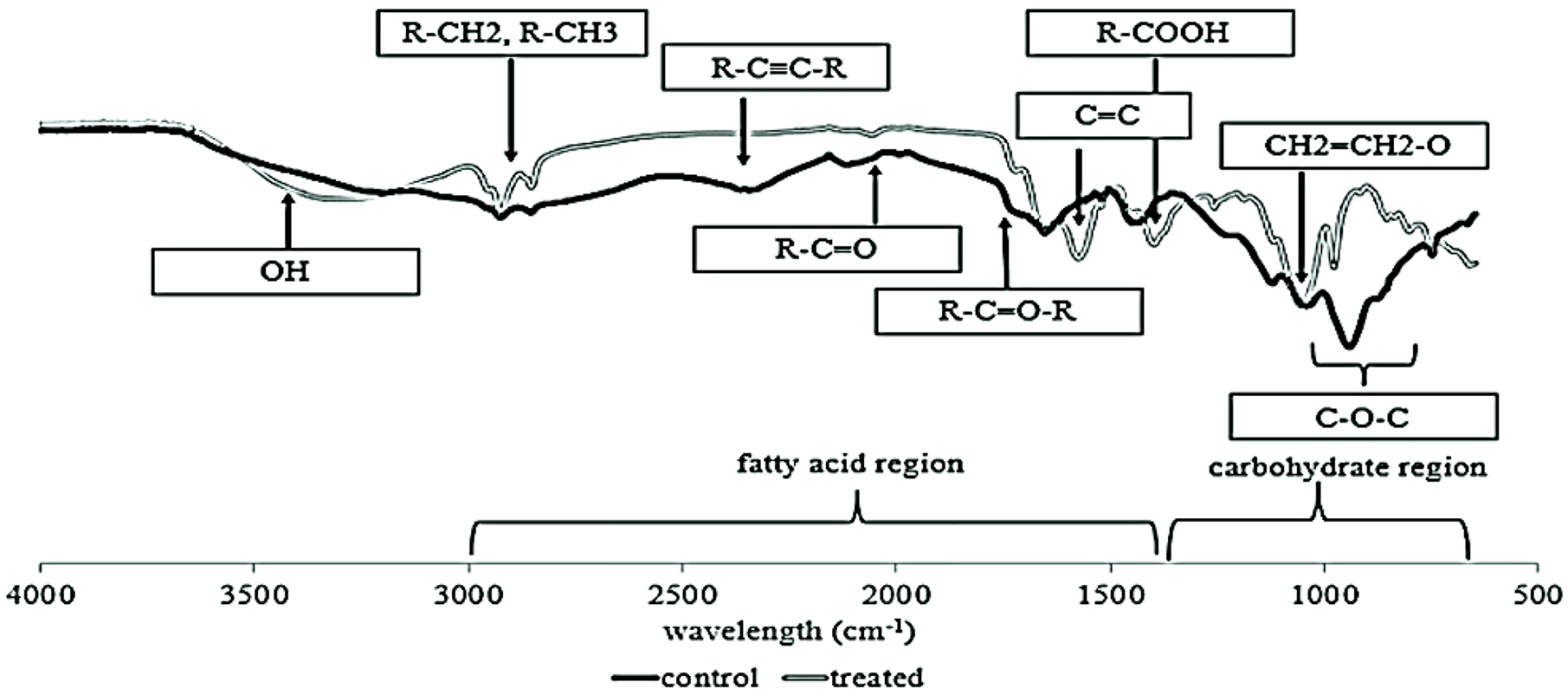
FTIR spectra of extracted, purified RL produced by *P. aeruginosa* RW9 in NB (control: dark-line) and NB with 10 mg l^-1^ of Cr (VI) (treated: grey-line) after 24 h of incubation.

The FTIR spectra of the pure RL provided more evidence supporting the glycolipid structure as RL (Fig. 5). Peaks corresponding to the characteristics of lipid and sugar moieties were evident within specific wavelength ranges, typically ranging from approximately 3,000 to 1,500 cm^-1^ for lipids and 1,400 to 700 cm^-1^ for sugars. Notably, a broad peak recorded at 3,700 to 3,200 cm^-1^ indicated the presence of the -OH functional group, while peaks at 2,926, 2,854, 2,345, and 2,100 cm^-1^ confirmed the presence of aliphatic -CH_2_ and -CH_3_ groups in the lipid moiety. Absorption bands around 1,738 and 1,456 cm^-1^ represented the C=O of carbonyl and carboxyl groups, respectively. Additionally, the presence of C-O stretching, and C-O-C of ester groups was observed in the 1,219 and 1,108 cm^-1^ ranges. Strong absorption at 1,045 cm^-1^ suggested the presence of rhamnose sugar, with stretching of C-O observed at absorption peaks of 943 and 750 cm^-1^. These spectral features aligned closely with those reported in prior studies [19].

The LC-MS spectra further supported the findings from NMR and FTIR analyses. Fragmentation analysis of the major components in both control and Cr (VI)-treated samples revealed characteristic daughter ions at specific m/z values. For instance, the first major component (m/z 649.37, Rha-Rha-C10-C10) displayed dominant daughter ions at m/z 143.00, 169.12, and 479.24 (see Table 2). Similarly, the second most abundant component, Rha-C10-C10 (m/z 502), exhibited prominent daughter ions at m/z 59.01, 169.12, 177.13, 303.19, and 339.25. These analyses confirmed the metabolite produced as a mixture of mono- and di-RL, as indicated by the characterization results.

### Effect of Cr (VI) on RL Congener Distribution

Cr (VI) exposure significantly impacted the congener distribution of the produced RL, as evidenced by Table 2. Statistical analysis using the T-test revealed a significant effect of 10 mg l^-1^ Cr (VI) on the ratio of di- to mono-RL produced (*p* < 0.05). In the control sample, predominant peaks corresponding to the molecular weight of eight RL homologs were observed. Mono-RL was detected at m/z values of 474, 503, 528, and 531, representing differences in fatty acid carbon length [16, 59]. Conversely, m/z values of 620, 649, 674, and 678 were recorded for di-RL. In the Cr(VI)-treated sample, the chromatogram displayed seven homolog peaks, with four being mono-RL and lacking a di-RL congener with m/z of 620 (Rha-Rha-C8-C10/Rha-Rha-C10-C8) [9]. This absence, consistent with commercial RL, confirms that Cr (VI) induced the synthesis of Rha-Rha-C8-C10/Rha-Rha-C10-C8. The relative abundance analysis revealed a slight decrease in the ratio of di-RL to mono-RL in Cr (VI)-treated cells (71.44 ± 0.04% di-RL: 28.53 ± 0.05% mono-RL) compared to control cells (73.36 ± 0.17% di-RL: 26.45 ± 0.05% mono-RL). This finding is consistent with previous research [59]. However, both control and Cr (VI)-treated cells exhibited the same dominant congener, Rha-Rha-C10-C10 (m/z 649), with relative abundances of 49.35% and 45.76%, respectively.

The high proportion of di-RL produced by *P. aeruginosa* RW9 (Table 2) is preferred for Cr removal due to their structural and functional advantages. Di-RL, characterized by two rhamnose units linked to a lipid chain, exhibits greater amphiphilicity compared to mono-RL. This property enhances their ability to form micelles and effectively interact with hydrophobic pollutants like Cr (VI). Di-RL demonstrates superior solubilization and emulsification capabilities, facilitating the sequestration of Cr (VI) ions from aqueous solutions and contaminated environments. Their higher surface activity stabilizes Cr (VI) in aqueous phases, thereby preventing its reduction to the more toxic Cr (III) form and improving removal efficiency. This functionality is crucial in microbial remediation, where RLs act as biosurfactants and metal chelators. Structurally, di-RLs can form stable complexes with metal ions through their dual rhamnose units, enhancing metal ion sequestration and reducing bioavailability. This property not only mitigates the toxic effects of heavy metals on microbial cells but also supports environmental remediation efforts by enhancing the specificity and efficiency of metal ion removal processes. Thus, the preference for di-RLs in Cr removal underscores their enhanced emulsifying properties, strong surface activity, and effective metal ion complexation ability, making them highly effective in bioremediation applications.

### Transcriptional Expression Study

**Fig. 6:**
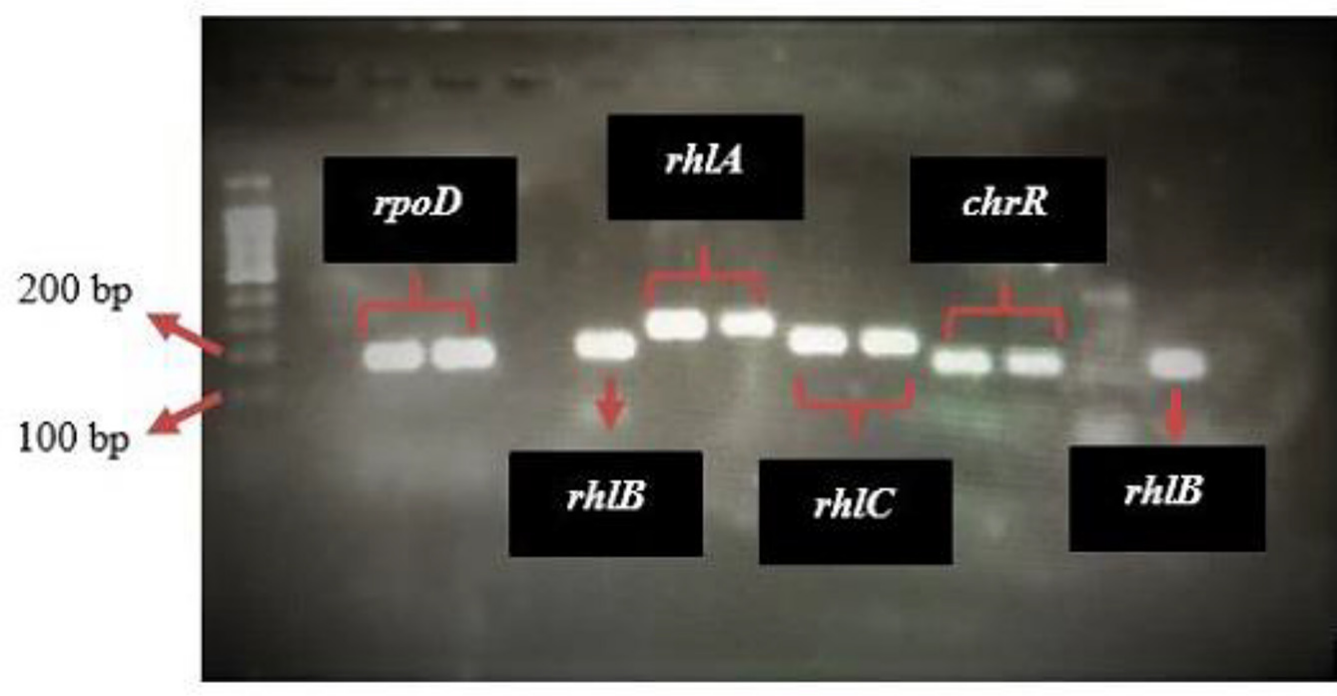
Gel image displaying PCR products for RL-encoded genes (*rhlA*, *rhlB*, and *rhlC*), reductase-encoded gene (*chrR*), and housekeeping gene (*rpoD*).

To elucidate the impact of Cr (VI) on RL biosynthesis, congener distribution, and reductase production, we assessed the transcriptional expression of RL-encoded genes (*rhlA*, *rhlB*, *rhlC*) and the reductase-encoded gene (*chrR*) using qRT-PCR. The gel image of the PCR products confirmed the presence of the target genes and the housekeeping gene, showing clear bands of the expected sizes: 260 bp (*rhlA*), 200 bp (*rhlB*), 150 bp (*rhlC*), 120 bp (*chrR*), and 150 bp (*rpoD*) (see Fig. 6). These findings align with previous studies [21, 26, 39]. Whole-genome sequencing (WGS) analysis revealed that *rhlA* and *rhlB* are located in a single operon, while *rhlC* is in a separate operon [25].

**Fig. 7:**
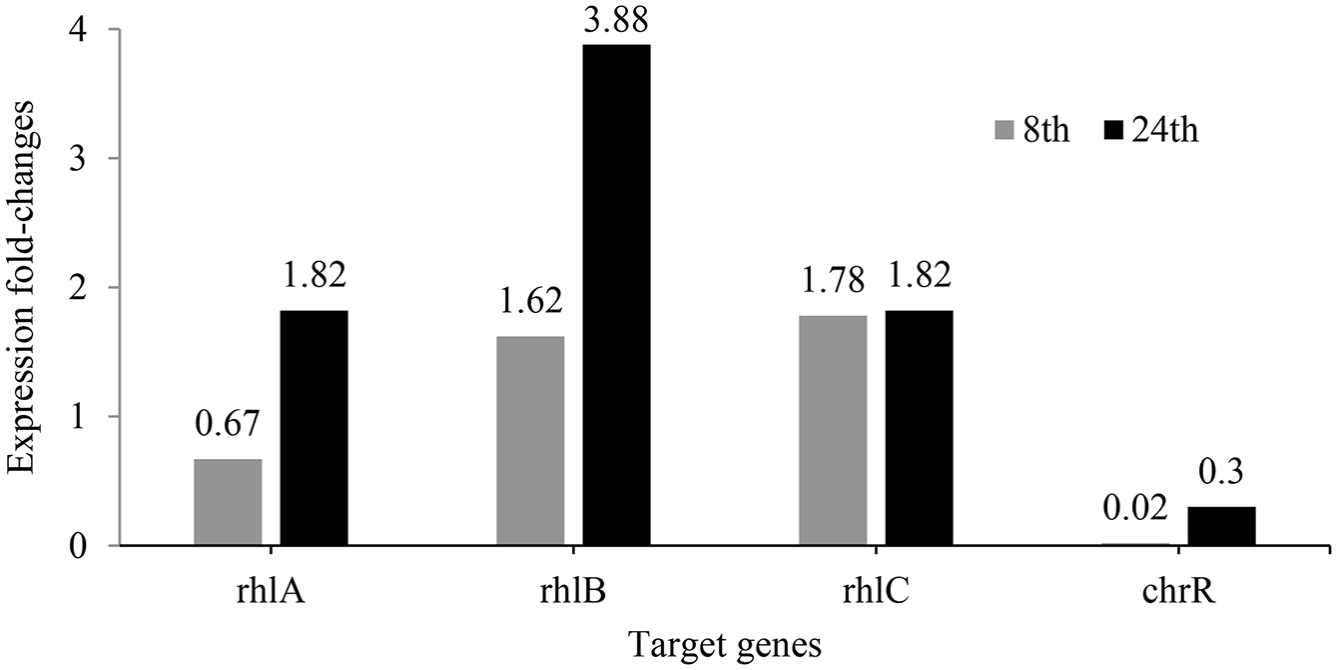
Transcriptional expression fold changes of each target gene in *P. aeruginosa* RW9. Gray columns represent samples from the 8^th^ h of incubation, while white columns represent samples from the 24^th^ h of incubation. Note: Fold-change was calculated from mean Ct values of duplicate reactions from triplicate samples at each sampling time, with RNA template concentration standardized at 200 ng per reaction. Target genes include RL-encoded genes (*rhlA*, *rhlB*, and *rhlC*) and the housekeeping gene *rpoD*.

Transcriptional expression of all target genes was lower at the 8^th^ h of incubation compared to the 24^th^ h (see Fig. 7). This upregulation might be attributed to oxidative stress resulting from Cr(VI) reduction [2], which activates the global regulon of the stationary phase sigma factor RpoS [43], responsible for regulating stress responses in *P. aeruginosa*. Bervoets and Charlier [7] reported that the *rhlAB* regulon, integrated with RpoS, is upregulated in response to environmental stressors such as oxidative stress. This study observed an increase in transcriptional expression of *rhlA* and *rhlB* by 1.82- and 3.88-fold at the 24^th^ h of incubation, respectively.

The lower induction of *rhlC*, evidenced by a modest fold change may initially suggest minimal changes over time, as noted in Fig. 7. However, this observation contrasts with the profound impact observed in RL congener distribution detailed in the LC-MS analysis in Table 2. This disparity can be attributed to the distinct operon locations of *rhlC* compared to *rhlA* and *rhlB*, resulting in varied expression levels and temporal dynamics. These differences prevent simultaneous enzyme production in a uniform stoichiometric ratio, thereby influencing RL congener distribution [1]. In addition to the oxidative stress caused by Cr (VI), the slight induction of *rhlC* over time might also reflect the role of RL to extracellularly sequester Cr (VI), thereby diminishing the likelihood of its ingress into the cells. This led to a reduced pool of intracellular Cr (VI) available for triggering the posttranscriptional regulation of RL, culminating in a decreased production of di-RL [39]. This finding consequently indicates a complex regulatory network influencing RL synthesis efficiency.

Moreover, it's noteworthy that previous studies under similar conditions with divalent ions like Cd(II) did not detect *rhlC* induction [39], highlighting the specificity and significance of *rhlC* induction in response to Cr(VI) exposure. Conversely, *chrR* exhibited low transcriptional expression levels at both sampling times, as depicted in Fig. 7. The minimal secretion of chromate reductase associated with this low expression likely limits its role in facilitating Cr (VI) extracellular sequestration.

### Future Research on Long-Term Viability in Cr (VI) Environments

This study has shown that *P. aeruginosa* RW9 strains are able to survive and thrive in habitats exposed to Cr (VI), which indicates their potential for long-lasting bioremediation efforts. The synthesis of metabolites such as RL plays a crucial function in the extracellular sequestration of Cr (VI), hence assisting in the bacteria's adaptive response. Nevertheless, prolonged exposure to Cr (VI) may impact the long-term viability of *P. aeruginosa* RW9. Prior research has shown that prolonged exposure of bacteria to Cr(VI) may result in alterations in gene expression involving amino acid metabolism, energy metabolism, and signal transduction mechanisms, as well as adaptation of cells to DNA damage [8, 32, 41, 56]. This adaptation is characterized by the overexpression of genes involved in DNA replication and repair, and the downregulation of genes linked with electron transport. In addition, it may include stress response regulators, metal resistance genes, or other adaptive processes, which further emphasize the long-term impacts of exposure to Cr (VI).

Even yet, while being based on the larger context of these long-term adaptive abilities, these results are mostly surface-level and do not delve deeply as they are focusing exclusively on instant responses. The subject remains largely unexplored, with barely any studies conducted to investigate this grey area. It is a well-established fact that bacteria, including species of *Pseudomonas*, can undergo adaptive modifications over time [32]. This process might include genetic mutations that either increase or reduce certain metabolic pathways, such as those related to the formation of RL and the decrease of Cr (VI). Hence, additional research is required to thoroughly evaluate such variables and attain an accurate picture of the long-term resilience and viability of *P. aeruginosa* strains in environments exposed to Cr (VI). This will enhance the practicality of bioremediation techniques.

## Conclusion

This study reveals a novel mechanism employed by *P. aeruginosa* RW9 for Cr(VI) bioremediation, focusing on the critical role of rhamnolipids (RL). Our research confirmed that RL is the primary metabolite involved in the reduction of Cr (VI) by *P. aeruginosa* RW9. Exposure to Cr (VI) resulted in a nearly threefold increase in RL yield, with over 70% of the RL mixture consisting of di-RL, which is highly beneficial for various downstream applications due to its efficient surface activity. Moreover, Cr (VI) exposure significantly upregulated the transcriptional expression of all RL-encoded genes. This high RL yield and the specific induction of *rhlC* are not commonly reported in studies focusing on divalent ions at comparable concentrations, highlighting the unique regulatory effect of Cr (VI) on the RL biosynthesis pathway. These findings indicate that Cr (VI) can be used to strategically enhance RL production, making it a potential micronutrient in RL production media. The ability of *P. aeruginosa* RW9 to produce high levels of RL, particularly di-RL, in response to Cr (VI) exposure suggests a promising application for chromium bioremediation. Moreover, the study's insights into the biochemical pathway regulation by Cr (VI) provide a foundation for optimizing RL yields, proposing the potential use of Cr (VI) as a micronutrient in media. This presented data carries substantial significance, understanding the interplay between Cr (VI) exposure and RL production dynamics to strategically manipulate production processes, ultimately improving the feasibility and efficiency of RL-based bioremediation strategies

## Supplemental Materials

Supplementary data for this paper are available on-line only at http://jmb.or.kr.



## Figures and Tables

**Table 1:** List of the primers used for each RL-encoded (*rhlA*, *rhlB*, *rhlC*) and housekeeping (*rpoD*) genes and their respective sizes and roles in the synthesis pathways of the metabolites.

Target genes	Primers	Sizes (bp)	Functions	References
*rhlA*	5’TGGACTCCAGGTCGAGGAAA 3’ (forward) 5’GAAAGCCAGCAACCATCAGC 3’ (reverse)	243	Synthesis of 3-(3-hydroxyalkanoyloxy) alkanoic acid (HAA) (biosurfactant precursor)	[26]
*rhlB*	5’GTCGAGTCCCTGGTTGAAGG 3’ (forward) 5’CGTGCTGGTGGTACTGTTCA 3’ (reverse)	191	Coding for Rhamnosyltransferase I	[39]
*rhlC*	5’ATCCATCTCGACGGACTGAC 3’ (forward) 5’GTCCAGGTCGTCGATGAAC 3’ (reverse)	158	Coding for the Rhamnosyltransferase II	[39]
*rpoD*	5’ GGG CGA AGA AGG AAA TGG TC 3’ (forward) 5’ CAG GTG GCG TAG GTG GAG AA 3’ (reverse)	158	Housekeeping	[39]

**Table 2:** Molecular weights, their structures, the molecular weight of dominant fatty acid moieties and relative abundance of RL by *P. aeruginosa* RW9 produced in control and 10 mg l^-1^ Cr(VI) treated experiments.

[M- H]^-^	Molecular structure	[M-H-C_m_H_2m+1_COOH]^-^	Relative abundance (%)
Control			
474	Rha-C8-C10	305	0.16
	Rha-C10-C8	333	
502	Rha-C10-C10	333	20.51
529	Rha-C10-C12:1	333	3.31
	Rha-C12:1-C10	359	
531	Rha-C10-C12	333	2.49
	Rha-C12-C10	361	
620	Rha-Rha-C8-C10	451	1.70
	Rha-Rha-C10-C8	479	
649	Rha-Rha-C10-C10	479	49.35
674	Rha-Rha-C10-C12:1	479	8.31
	Rha-Rha-C12:1-C10	505	
678	Rha-Rha-C10-C12	479	3.28
	Rha-Rha-C12-C10	507	10.91
Cr(VI)-treated			
474	Rha-C8-C10	305	1.05
	Rha-C10-C8	333	
503	Rha-C10-C10	333	21.34
528	Rha-C10-C12:1	333	3.51
	Rha-C12:1-C10	359	
531	Rha-C10-C12	333	2.63
	Rha-C12-C10	361	
649	Rha-Rha-C10-C10	479	45.76
674	Rha-Rha-C10-C12:1	479	9.10
	Rha-Rha-C12:1-C10	505	
676	Rha-Rha-C10-C12	479	16.58
	Rha-Rha-C12-C10	505	
