# Supplementary material for: Novel Insights into Cr(VI)-Induced Rhamnolipid Production and Gene Expression in Pseudomonas aeruginosa RW9 for Potential Bioremediation
Source: J Microbiol Biotechnol. 2024 Jul 19;34(9):1877–89. doi: 10.4014/jmb.2406.06034 (PMC11473487; doi:10.4014/jmb.2406.06034)
Supplement: Supplementary file 1 [file jmb-34-9-1877-supple.pdf]

**J. Microbiol. Biotechnol. 2024. 34(9): 1877–1889**
**https://doi.org/10.4014/jmb.2406.06034**

# Supplementary Figure

## Novel insights into Cr(VI)-induced rhamnolipid production and gene expression in *Pseudomonas aeruginosa* RW9 for potential bioremediation

Fatini Mat Arisah[1], Norhayati Ramli[1,2], Hidayah Ariffin[1,2], Toshinari Maeda[3], Mohammed Abdillah Ahmad Farid[3], and Mohd Zulkhairi Mohd Yusoff[1,2]*

[1] Department of Bioprocess Technology, Faculty of Biotechnology and Biomolecular Sciences, Universiti Putra Malaysia, Serdang 43400, Selangor, Malaysia

[2] Laboratory of Biopolymer and Derivatives, Institute of Tropical Forestry and Forest Products (INTROP), Universiti Putra Malaysia, 43400 UPM Serdang, Selangor, Malaysia

[3] Department of Biological Functions Engineering, Graduate School of Life Science and Systems Engineering, Kyushu Institute of Technology, 2-4 Hibikino, Wakamatsu-ku, Kitakyushu 808-0196, Japan

***Corresponding author:** Mohd Zulkhairi Mohd Yusoff / **E-mail** mzulkhairi@upm.edu.my

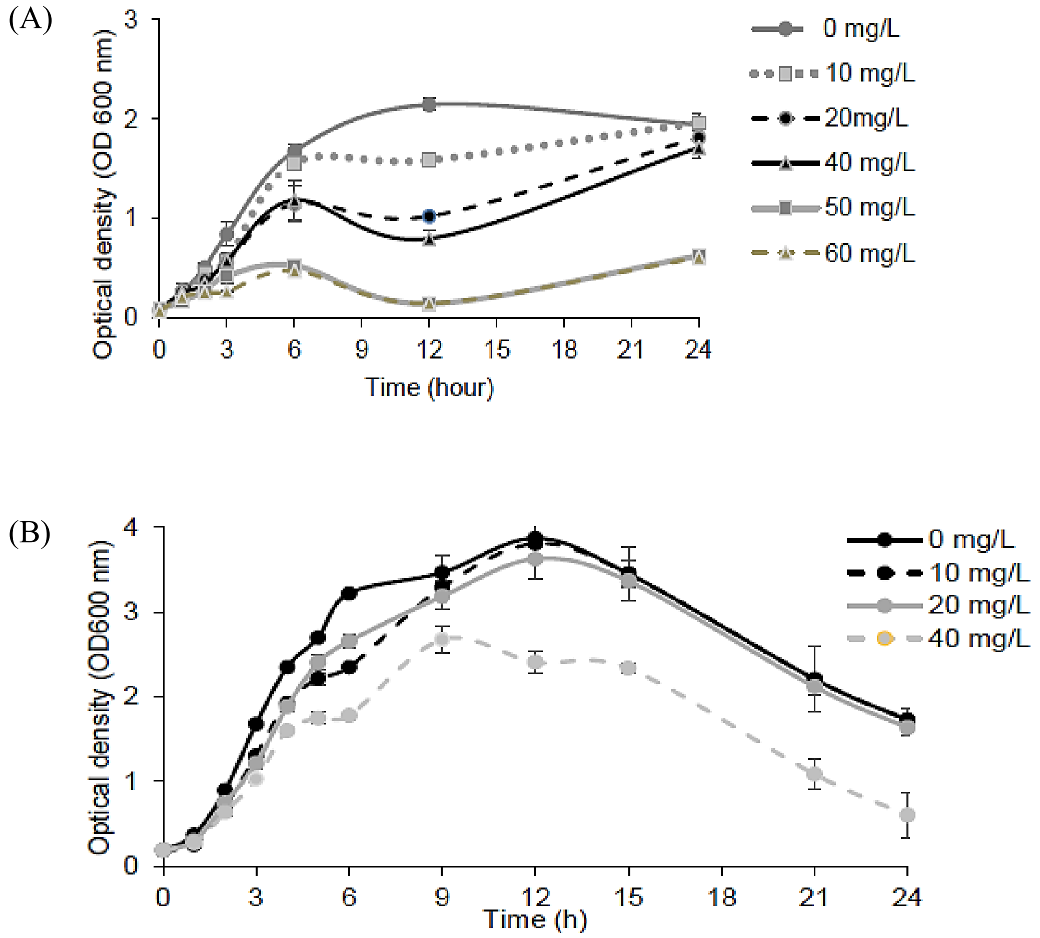


**Fig. S1. Growth profile of (A)** *P. aeruginosa* RW9 and **(B)** *Bacillus* sp. L14 cultivated in NB with different concentrations of Cr(VI) at 150 rpm agitation, 30 °C. Note: *Bacillus* sp. L14 enters the lysis phase after 18 h of incubation (1).

**Reference**

1. Özgür K. and Nilüfer C. 2011. Isolation of protease producing novel *Bacillus cereus* and detection of optimal conditions. *African J. Biotechnol*. **10,** 1160–1164.
